# Stellate Ganglion Block Relieves Long COVID-19 Symptoms in 86% of Patients: A Retrospective Cohort Study

**DOI:** 10.7759/cureus.45161

**Published:** 2023-09-13

**Authors:** Lisa Pearson, Alfred Maina, Taylor Compratt, Sherri Harden, Abbey Aaroe, Whitney Copas, Leah Thompson

**Affiliations:** 1 Pain Management, University of South Florida, Tampa, USA; 2 Pain Management, Metamorphosis Ltd., Canon City, USA; 3 Pain Management, Texas Christian University, Fort Worth, USA; 4 Anesthesia, Missouri State University, Springfield, USA; 5 Pain Management, Metamorphosis Ltd., Colorado Springs, USA; 6 Nursing, University of Colorado, Denver, USA

**Keywords:** autonomic nervous system modulation, long covid-19, long covid syndrome, post-covid autonomic dysfunction, stellate ganglion block, stellate ganglion block (sgb), treatment for long covid

## Abstract

Post-COVID-19 condition, also known as long COVID-19 syndrome and post-acute sequelae of SARS-CoV-2, encompasses an array of symptoms that persist well beyond the initial phase of the viral infection. These symptoms can range in intensity, from mild and manageable to severe and incapacitating. Due to the evolving nature of the SARS-CoV-2 pandemic, treatment protocols for the illness are in a constant state of evolution. The early stage of long COVID-19 syndrome contributes to a dearth of treatment protocols based on empirical evidence, while the absence of a conclusive pathophysiological understanding further complicates the development of such protocols. Current treatment regimens include homeopathic medicine, specialist system-focused treatments, infusion therapies, hyperbaric oxygenation, antivirals, and polypharmacy. The physiological, psychological, and societal impact of long COVID-19 cannot be approached casually and must govern the intensity with which the healthcare community approaches the treatment of long COVID-19 syndrome.

In this 41-patient cohort study from a chronic pain management practice, the use of either unilateral or bilateral stellate ganglion block (SGB) was explored to manage symptoms associated with long COVID-19 syndrome. Results indicated that a substantial proportion of patients (86%) experienced a reduction of their symptoms following SGB treatment.

## Introduction

Long COVID-19 encompasses a range of lingering symptoms following an acute COVID-19 infection. Defined by the Centers for Disease Control and Prevention (CDC) as symptoms that persist for three or more months post-infection without previous existence [1], data from the U.S. Census Bureau, when analyzed by the CDC's National Center for Health Statistics (NCHS), show about 7.5% of US adults suffer from these symptoms [1]. Studies have found post-COVID-19 symptoms in 15.1% of people 12 months after the initial infection, with prevalence rates sometimes as high as 91% [2-4].

The CDC lists numerous symptoms of long COVID-19, such as fatigue, exacerbated symptoms after physical or mental exertion, respiratory problems, cognitive issues, sleep disturbances, gastrointestinal problems, and changes in menstrual cycles, among others [1]. Zhang et al. used machine learning in a study with 34,605 participants to analyze over 137 symptoms, categorizing them into four distinct sub-phenotypes [5]. The reproducible sub-phenotypes are (1) cardiac and renal, (2) respiratory, sleep, and anxiety, (3) musculoskeletal and nervous system, (4) and digestive and respiratory systems. This classification could assist in the development of future protocols tailored to specific sub-phenotypes, given that varying pathophysiological alterations could be influenced by disparate mechanisms, which may serve as potential therapeutic targets.

The pathophysiology behind long COVID-19 remains a topic of study, but evidence points toward the nervous system's immunomodulatory activities playing a role in symptom manifestation. Several studies have shown links between long COVID-19 and an imbalanced immune response with persistent inflammation. Increased levels of cytokines such as interleukin (IL)-6, tumor necrosis factor-alpha (TNF-α), IL-1β, IL-8, and IL-10 suggest a sustained inflammatory state [6]. The C-reactive protein, which indicates inflammation, is elevated in long COVID-19 patients [7]. Its levels correlate with symptom severity and duration, but there is variability among individuals [6-8].

Post-viral symptoms resembling dysautonomia have been reported for various viruses [4]. A subset of dysautonomia, postural orthostatic tachycardia syndrome (POTS), displays several symptoms that overlap with long COVID-19. The incidence of post-COVID-19 dysautonomia is noted at 2.5% [4], which might be an underestimate given the shared symptoms. The sympathetic nervous system's role in long COVID-19 is evident, implying possible therapeutic targeting.

Historically, the nervous and immune systems were thought to function independently. However, research by Elenkov et al. [8] and Tracey [9] has shown their interdependence. Fischer posits that during viral infections like COVID-19, the autonomic nervous system (ANS) plays a crucial role in managing acute hyperinflammation, endothelial dysfunction, and other related issues [10]. A malfunctioning ANS, marked by sympathetic hyperactivity, can be observed during these infections [11].

For long COVID-19, the sympathetic system's overactivity coupled with an underactive vagus nerve might allow persistent inflammation. This ongoing inflammation is possibly central to the characteristic symptoms of long COVID-19, as it disrupts the balance between the sympathetic and parasympathetic systems.

Given our understanding of the significant autonomic component in long COVID-19, our intervention targeted the sympathetic nervous system, using a stellate ganglion block (SGB) to potentially alleviate long COVID-19 symptoms.

A version of this article was previously posted on a pre-print server (medRxiv) on August 15, 2023.

## Materials and methods

Study design

A cohort population that consisted of self-referred patients who received an SGB for the treatment of long COVID-19 symptoms in our Colorado clinics between September and December of 2022. The patients had experienced symptoms of long COVID-19 for a duration ranging from three to 29 months. The treatment consisted of an ultrasound-guided injection around the stellate ganglion.

Sampling

Patients who responded to our post-procedure follow-up via phone call, in person, or telehealth meeting.

Data collection

Data were gathered and stored in an Excel spreadsheet (Microsoft Corporation, Redmond, WA). The data points included 18 items from the CDC list of the most prevalent symptoms associated with long COVID-19 syndrome. For each patient, the data indicated the presence of specific symptoms before the SGB procedure, and whether these symptoms improved or remained unchanged after the SGB.

Variables and measurements

Symptoms evaluated included difficulty breathing or shortness of breath, cough, tiredness/fatigue, chest or stomach pain, joint or muscle pain, tachycardia/palpitations, symptoms that get worse after physical activity, pins and needles feeling, diarrhea, change in smell and taste, fever, dizziness/lightheadedness when standing, difficulty sleeping, rash, mood changes, headache, changes in menstrual cycle, brain fog/confusion, age, and gender. Symptoms were measured as present or not present before SGB and improved or unimproved after SGB.

Data analysis

SPSS version 28.0.1.0 (IBM Corp., Armonk, NY) was used for data analysis with paired sample proportions.

Ethical considerations

All patients included in this study signed an informed consent for the procedure and subsequent collection of de-identified data.

## Results

The treatment outcomes of 41 patients (18 male and 23 female) with an age range between 18 and 89 years who underwent SGB for long COVID-19 syndrome were evaluated. The population consisted of patients from the United States who received treatment in our clinics between September and December of 2022 and responded to our post-procedure follow-up calls. The timeframe between the procedure and the data collection was variable depending on patient response times. The evaluation of the patients' long COVID-19 symptoms was based on the CDC list of the most prevalent symptoms of the condition.

The initial symptoms reported by the 41 patients included shortness of breath (41%, 10 males and seven females), cough (24%, six males and four females), fatigue (85%, 15 males and 20 females), chest pain (24%, four males and six females), joint/muscle pain (39%, seven males and nine females), tachycardia or palpitations (22%, four males and five females), post-exertional malaise (66%, 14 males and 13 females), pins and needles sensation (22%, three males and six females), diarrhea (20%, three males and five females), changes in taste and smell (44%, 10 males and eight females), fever (2%, one female), dizziness (41%, 11 males and six females), difficulty sleeping (34%, seven males and seven females), rash (5%, two females), mood changes (51%, 11 males and 10 females), headache (39%, eight males and eight females), changes in the menstrual cycle (12%, five females), and brain fog (80%, 15 males and 18 females) (Table 1).

**Table 1:** This table shows the prevalence of long COVID-19 symptoms reported by patients who underwent stellate ganglion block treatment for long COVID-19 syndrome

Symptom	Total reports	Percentage	Males	Females
Shortness of breath	17	41%	10	7
Cough	10	24%	6	4
Fatigue	35	85%	15	20
Chest pain	10	24%	4	6
Joint/muscle pain	16	39%	7	9
Tachycardia/palpitations	9	22%	4	5
Post-exertional malaise	27	66%	14	13
Pins and needles	9	22%	3	6
Diarrhea	8	20%	3	5
Changes in taste/smell	18	44%	10	8
Fever	1	2%	0	1
Dizziness	17	41%	11	6
Difficulty sleeping	14	34%	7	7
Rash	2	5%	0	2
Mood changes	21	51%	11	10
Headache	16	39%	8	8
Changes in menstrual cycle	5	12%	0	5
Brain fog	33	80%	15	18

The dataset, as illustrated in Table 2, presents an assessment of the treatment efficacy in mitigating symptoms. The data indicates noteworthy amelioration in symptoms such as cough, fatigue, and joint pain. There is a substantial proportion of individuals reporting relief, with notable variations in improvement rates between males and females for specific symptoms. The findings demonstrate that a substantial proportion of patients (86%) reported a reduction in at least some of their symptoms. Out of the 35 individuals who experienced symptom relief, a majority of 25 patients (61% of the total 41 patients) reported relief from all the long COVID-19 symptoms they initially presented with (Table 2).

**Table 2:** This table displays the percentage of patients who reported resolution of specific long COVID-19 symptoms following stellate ganglion block treatment

Symptom	Total reports	All relief (%)	Males (relief %)	Females (relief %)
Shortness of breath	17	88	80	100
Cough	10	80	80	67
Fatigue	35	77	67	85
Chest pain	10	80	50	100
Joint pain	16	94	86	100
Tachycardia/palpitations	9	78	50	100
Symptoms worsen	27	74	64	92
Pins and needles	9	100	100	100
Diarrhea	8	88	67	100
Changes in taste/smell	18	56	40	75
Fever	1	100	-	100
Dizziness	17	77	64	100
Difficulty sleeping	14	71	57	86
Rash	2	100	-	100
Mood changes	21	76	64	90
Headache	16	81	63	100
Changes in menstrual cycle	5	80	-	80
Brain fog	33	79	60	94

**Figure 1:**
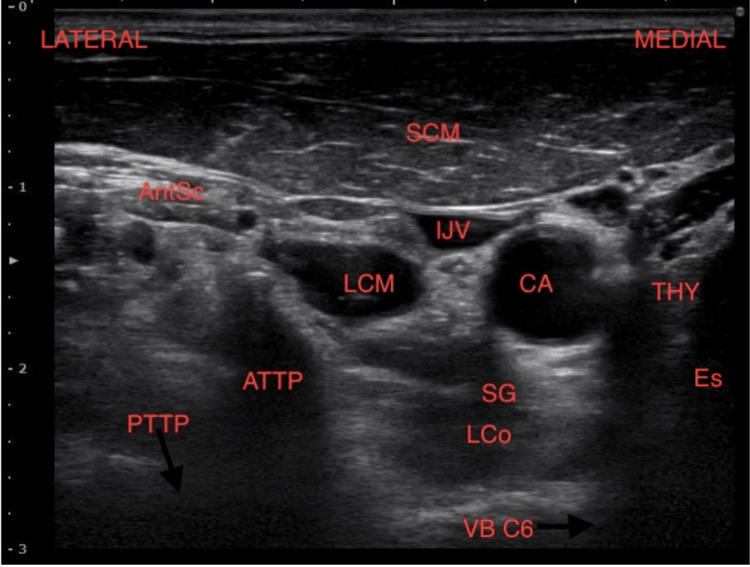
Ultrasound image of structures of interest AntSc: anterior scalene; SCM: sternocleidomastoid muscle; ATTP: anterior transverse process; PTTP: posterior transverse process; LCo: longus colli muscle; ITA: inferior thyroid artery; IJV: internal jugular vein; CA: carotid artery; SG: location for stellate block; Thy: thyroid; Es: esophagus; VB C6: C-6 vertebral body.

Ultrasound-guided SGBs were performed with patients lying in a supine position with the head of the bed slightly elevated and turned away from the side of the procedure. This positioning, combined with ultrasound guidance, reduces the risk of unintended outcomes, such as pneumothorax, vascular puncture, or cervical nerve intrusion. Elevating the head of the bed helps decompress the subclavian vessels and turning the head away from the procedure side shortens the distance between the needle entry point and the stellate ganglion. The blocks were performed following standard aseptic procedures. The cricothyroid notch, the border of the sternocleidomastoid muscle, and Chassaignac's tubercle serve as useful anatomical landmarks during the procedure. A skin marker was used to mark the expected placement of the ultrasound prior to application. The ultrasound probe was positioned transversely at the level of the cricothyroid notch, and the structures of concern were identified: trachea, esophagus, thyroid gland, blood vessels, muscles, and nerves (Figure 1). A lateral to medial in-plane approach was utilized to avoid the structures of concern and to facilitate ideal needle tip placement. Located anterolaterally to the longus colli muscle, deep to the prevertebral fascia, and superficial to the fascia investing the longus colli muscle. A skin wheal with 1 ml of 1% lidocaine with sodium bicarbonate 8% was made, followed by the introduction of a 2-inch or 4-inch echogenic needle (size determined by body habitus) with continuous ultrasound guidance to the area of the stellate ganglion. A mixture of 2 ml 2% chloroprocaine, 2 ml 1% lidocaine, and 1 ml 0.2% ropivacaine was used as the injectate, and real-time ultrasound monitoring was used to ensure proper deposition around the stellate ganglion [12-15].

This technique did not result in any significant adverse events. Three patients experienced a vagal response, all of which happened immediately after a left-sided block and resolved quickly with reverse Trendelenburg positioning. Transient hoarseness occurred in four patients and resolved upon dissipation of the local anesthetic, which usually occurred within an hour.

Patients who live locally received a right-sided SGB unless their symptoms were purely cardiac in nature. Patients who traveled from out of state, first received a right SGB, followed by a left-sided block once the Horner's syndrome (drooping eyelid, pupil constriction, and absence of sweating) from the previous block had resolved.

The resolution of long COVID-19 symptoms following SGB was found to occur rapidly, with most patients reporting a noticeable improvement in symptoms within 15 minutes of the block. The resolution of symptoms that could not be clearly evaluated immediately after the SGB (brain fog, fatigue) was observed to occur at different time points, with some patients reporting a steady improvement over one to two weeks, while others reported that their symptoms resolved within a day. However, it should be noted that the current study did not specifically evaluate the time-based resolution of symptoms.

The symptoms most frequently reported by our patients were fatigue, observed in 85% of cases, and brain fog, present in 80% of individuals. These symptoms showed the highest response to treatment, with 77% of patients experiencing fatigue reporting significant relief, and 80% of those suffering from brain fog reporting relief. In our patient group, the loss of taste and smell proved to be the most challenging symptom to treat, with only 56% of patients reporting improvement. Although, we were able to obtain a higher response rate for anosmia with the addition of a trigeminal nerve block (Table 2).

There was a marked statistical significance across all symptoms in response to SGB treatment, with p-values below 0.001 for most symptoms, demonstrating the broad effect of the treatment. The exception was “changes in menstruation,” for which the significance was still noteworthy with a p-value less than 0.05, in both one-sided and two-sided tests. This suggests a comprehensive impact of the SGB treatment across a range of symptoms (Table 3).

**Table 3:** Paired samples test of significance for each symptom

	Test type	Difference in proportions	Asymptotic standard error	Z	Significance	
					One-sided p	Two-sided p
Pair 1: Difficulty breathing or shortness of breath - Difficulty breathing or shortness of breath post treatment	McNemar	.366	.075	3.873	.001	.001
Pair 2: Cough - Cough post treatment	McNemar	.195	.062	2.828	.002	.005
Pair 3: Fatigue or tiredness - Fatigue or tiredness post treatment	McNemar	.659	.074	5.196	.001	.001
Pair 4: Chest or stomach pain - Chest or stomach pain post treatment	McNemar	.195	.062	2.828	.002	.005
Pair 5: Joint or muscle pain - Joint or muscle pain post treatment	McNemar	.366	.075	3.873	.001	.001
Pair 6: Tachycardia or palpitations - Tachycardia or palpitations post treatment	McNemar	.171	.059	2.646	.004	.008
Pair 7: Symptoms that get worse after physical or mental activities - Symptoms that get worse after physical or mental activities post treatment	McNemar	.512	.078	4.583	.001	.001
Pair 8: Pins and needles feeling - Pins and needles feeling post treatment	McNemar	-.780	.065	-5.657	.001	.001
Pair 9: Diarrhea - Diarrhea post treatment	McNemar	.171	.059	2.646	.004	.008
Pair 10: Changes in taste or smell - Changes in taste or smell post treatment	McNemar	.244	.067	3.162	.001	.002
Pair 11: Fevers - Fevers post treatment	McNemar	-.976	.024	-6.325	.001	.001
Pair 12: Dizziness of lightheadedness when standing - Dizziness of lightheadedness when standing post treatment	McNemar	.317	.073	3.606	.001	.001
Pair 13: Difficulty sleeping - Difficulty sleeping post treatment	McNemar	.244	.067	3.162	.001	.002
Pair 14: Rash - Rash post treatment	McNemar	-.951	.034	-6.245	.001	.001
Pair 15: Mood changes - Mood changes post treatment	McNemar	.390	.076	4.000	.001	.001
Pair 16: Headache - Headache post treatment	McNemar	.317	.073	3.606	.001	.001
Pair 17: Brain fog or confusion - Brain fog or confusion post treatment	McNemar	.634	.075	5.099	.001	.001
Pair 18: Changes in menstruation - Changes in menstruation post treatment	McNemar	.098	.046	2.000	.023	.046

A gender disparity was observed in both the initial presentation of symptoms and symptom resolution. Women reported a higher frequency of symptoms related to long COVID-19; however, they also demonstrated a higher rate of symptom resolution (Table 3).

Only two patients experienced a return of symptoms as of September 2023. For one, this relapse was linked to another viral illness occurring three months after their initial stellate procedure. The cause of symptom return for the second patient remains unclear.

## Discussion

The precise mechanism through which SGB ameliorates long COVID-19 symptoms is not fully understood. Fischer et al. theorized that the immune and inflammatory responses during the acute phase of long COVID-19 and the ensuing post-acute symptoms were due to a malfunction in the ANS, particularly sympathetic hyperactivity [10]. They argued that SGB, by modulating the ANS, could disrupt overactive processes within nerve-immune-inflammation feedback loops, enabling their self-regulation. Furthermore, the impact of SGB went beyond the ANS, influencing downstream regulatory actions, including immune and inflammatory system control, cytokine production, endothelial function, and microcirculatory function regulation [10]. SGB has been utilized for a broad spectrum of conditions, including pain syndromes and disorders of the immune and endocrine systems. Lipov et al. suggested that the therapeutic action of SGB might primarily involve sympathetic block-mediated peripheral vasodilation [16]. However, given the diversity of SGB applications, they acknowledged that the mechanism might be more multifaceted. Deng et al. demonstrated multiple changes in the neuroendocrine system of rats that received an SGB prior to surgery [17]. The changes included reductions in neuronal loss, decreased microglial activation, and reductions in inflammatory markers such as TNF-α, IL-1β, and IL-6. Others hypothesized that SGB resulted in changes in voltage-gated sodium channels of peripheral nerves and the central response by spinal feedback loops, thus decreasing symptoms [4]. Neuronal growth factor was also suspected to play a role in the effects of an SGB [18].

Numerous research studies have demonstrated the effects of SGB in the modulation of the immune system and hyperinflammation. These effects include the reduction of natural killer cell activity [19,20], as well as the decrease in levels of inflammatory cytokines such as IL-1, IL-4, IL-6, IL-8, and TNF-α, and the concurrent increase of anti-inflammatory cytokine IL-10 and calcitonin gene-related peptide [21-23]. Additionally, SGB has been shown to regulate endothelial dysfunction [24], microcirculation, and coagulopathy [20,25,26]. Furthermore, it contributes to the reduction of neurogenic pulmonary edema [15,24,27] and pulmonary arterial hypertension [28]. Another notable effect is the reduction of pathological positive feedback loops [10,29,30].

The reversal of anosmia (loss of smell) and dysgeusia (altered taste) post SGB aligns with the restoration of parasympathetic function due to sympathetic blockade of the stellate ganglion, the conduit for all sympathetic innervation of the head and neck. It is plausible to infer that sympathetic hyper-reactivity also triggers anosmia and dysgeusia by interrupting sympathetic outflow to cranial nerves specifically cranial nerve (CN) I (olfactory), CN VII (facial), and CN IX (glossopharyngeal) [14].

The timing for employing SGB in managing long COVID-19 syndrome may be important, as the degree of neuroadaptation that has occurred may decrease the effectiveness of the SGB. Our clinical observations indicate that delayed SGB, such as in individuals with long-standing analogous post-viral ailments (Lyme disease, chronic fatigue syndrome, and myalgic encephalopathy), exhibited notably diminished responsiveness to SGB as compared to long COVID-19 patients. Achieving only a 10-20% reduction in their symptoms with each injection.

Frequently reported side effects include hoarseness, attributed to the anesthetic’s effect on the recurrent laryngeal nerve, and manifestations of Horner's syndrome, which includes facial anhidrosis, ptosis, and miosis on the side of the injection. Additionally, patients may report a localized warmth sensation or even dysphagia, the latter due to transient esophageal compromise. While less prevalent, there are notable complications such as systemic effects from inadvertent anesthetic vascular uptake leading to seizures, respiratory compromise stemming from phrenic nerve involvement, hypersensitivity reactions to the agents employed, localized hematoma formation, particularly in patients on anticoagulant therapy, post-procedural infections, unintentional vascular injection, neuropathic sequelae, transient hypertension, and in rare instances, inadvertent spinal or epidural drug administration with its concomitant neurological implications.

In our practice, SGB has proven beneficial in treating long COVID-19 symptoms, and we urge its prompt consideration to avert possible diminished effectiveness due to time-sensitive neuroadaptive changes.

This study's results, based on responses from 41 of 60 treated patients, may not wholly represent all patient experiences. Those with less positive outcomes might have opted out of participating.

The choice of local anesthetic could influence SGB efficacy. We used a mix of chloroprocaine, lidocaine, and ropivacaine. Lidocaine is known for its immunomodulatory properties, while others have not been tested in this context. Further research might help determine the most effective local anesthetic and the underlying therapeutic mechanisms of SGB in long COVID-19 patients.

The use of ultrasound guidance during SGB injections is advocated; in our practice, no serious adverse events were reported from SGB injections performed under ultrasound guidance. We believe that the risk profile of SGB shifts when using ultrasound guidance compared to fluoroscopy. Ultrasound allows for real-time visualization of the target structure and surrounding anatomy, potentially reducing the risks of vascular injection and damage to adjacent structures. Moreover, the avoidance of ionizing radiation with ultrasound is another advantage over fluoroscopy. However, the proficiency of the practitioner in ultrasound techniques is paramount, as suboptimal imaging or interpretation can negate these advantages. We believe that overall ultrasound-guided SGB, when executed skillfully, can offer a safer and more precise procedural alternative.

In our clinical practice, right-sided SGBs were the most frequently performed procedures. Bilateral SGBs were reserved for patients who were coming from out-of-state or experiencing predominately dysautonomia related to long COVID-19 syndrome. The physiological effects of SGBs can vary depending on the side of the injection. Right-sided SGBs have been shown to result in greater sympathetic blockade than left-sided SGBs, leading to a greater decrease in heart rate and blood pressure, likely due to the right stellate ganglion's dominance and more direct connection to the heart via the cardiac plexus [9,18]. In contrast, left-sided SGBs produce a greater degree of parasympathetic effect than right-sided SGBs, leading to a greater increase in heart rate and cardiac output, likely due to the left vagus nerve's stronger influence on the heart and its direct connection to the sinoatrial node [9,18]. While no data were collected to analyze the relationship between laterality and patient response, we believe it is important to investigate the optimal laterality for administering SGBs based on specific symptoms of long COVID-19.

Six patients failed to experience symptom relief, and further evaluation revealed that three had rare disorders that were contributing to their symptoms. It is noteworthy that these diagnoses were uncommon, leading us to question if there may be undiagnosed underlying medical conditions in some of the remaining patients who were unresponsive to treatment.

Limitations

The limited sample size and potential bias of only having the patients who completed post-procedural follow-up are limitations of this study.

## Conclusions

The results of our cohort study indicate that SGB is a promising treatment option for patients suffering from long COVID-19 syndrome. The rapid resolution of symptoms and high success rate of treatment make SGB a compelling treatment option for long COVID-19 patients. However, it should be noted that our findings are limited to a specific subset of patients, and further research is needed to confirm these results. The use of ultrasound guidance and the specific anesthetic used may also play a role in the efficacy of SGB, and further studies in these areas are warranted.

While further double-blind placebo-controlled testing is necessary, the highly favorable patient response to this treatment suggests that it should not be withheld by healthcare providers. Despite the need for additional studies, we believe that this safe and effective procedure should be made broadly available to patients suffering from long COVID-19.
