# Correction: Stellate Ganglion Block Relieves Long COVID-19 Symptoms in 86% of Patients: A Retrospective Cohort Study

**DOI:** 10.7759/cureus.c295

**Published:** 2025-09-16

**Authors:** Lisa Pearson, Alfred Maina, Taylor Compratt, Sherri Harden, Abbey Aaroe, Whitney Copas, Leah Thompson

**Affiliations:** 1 Pain Management, University of South Florida, Tampa, USA; 2 Pain Management, Metamorphosis Ltd., Canon City, USA; 3 Pain Management, Texas Christian University, Fort Worth, USA; 4 Anesthesia, Missouri State University, Springfield, USA; 5 Pain Management, Metamorphosis Ltd., Colorado Springs, USA; 6 Nursing, University of Colorado, Denver, USA

The Ethics Statement and Conflict of Interest Disclosures section has been updated to disclose the following:

**Financial relationships:** All authors declare(s) employment from Metamorphosis Pain Management. The procedures described in this study were performed at Metamorphosis Pain Management.

